# Comparison of the Laboratory Standard Washing Using CIPAC Washing Agent and the Domestic Washing on Three Recommended Types of Long-Lasting Insecticidal Mosquito Nets

**DOI:** 10.1371/journal.pone.0074824

**Published:** 2013-10-09

**Authors:** Jean Pierre Nabléni Ouattara, Johanna Louwagie, Olivier Pigeon, Pieter Spanoghe

**Affiliations:** 1 Laboratory of Crop Protection Chemistry, Department of Crop Protection, Ghent University, Gent, Belgium; 2 Department of Textiles, Ghent University, Zwijnaarde, Belgium; 3 Walloon Agricultural Research Centre (CRA-W), Agriculture and Natural Environment Department, Plant Protection Products and Biocides Physico-Chemistry and Residues Unit, Gembloux, Belgium; 4 Laboratoire National de Santé Publique (LNSP), Boulevard des Tansoba kiéma, Burkina Faso; Centers for Disease Control and Prevention, United States of America

## Abstract

**Background:**

One of the best ways to prevent malaria is the use of insecticide-treated bed nets. Manufacturers pursue easier, safer and more efficient nets. Hence, many studies on the efficacy and wash resistance using World Health Organization standards have been reported. The commonly used detergent is “Savon de Marseille”, because it closely resembles actually used soaps. At the 54^th^ Collaborative International Pesticides Analytical Council (CIPAC) Technical Meeting in 2010, it was suggested to replace it by a standardized “CIPAC washing agent”. The aim of this study was to investigate the difference between a laboratory hand washing simulation using the CIPAC washing agent (method-1) and a domestic washing (method-2) on different bed nets, as well as the effect of the drying process on the release of active ingredient.

**Methods:**

Interceptor®, Permanet®2.0 and Netprotect® nets were used in three treatments, each repeated 20 times. The first treatment included method-1 washing and indoor drying. The second treatment included method-2 washing and indoor drying. The third treatment used method-2 washing and UV-drying. The residual insecticide contents were determined using gas chromatography.

**Results:**

The washing procedure and the number of washes have a significant effect on the release of active ingredient. Statistically, the two washing methods have the same effect on removing the active ingredient from the Interceptor® and Permanet®2.0 net, but a significantly different influence on the Netprotect® nets. The drying process has no significant effect on the insecticide.

**Conclusion:**

Both washing procedures affected the amount of insecticide remaining on nets independently of the impregnation technology. The active ingredient decreases with the number of washing cycles following an exponential or logarithmic model for coated nets. The laboratory hand washing simulation had more impact on the decrease of active ingredient content of the Netprotect® nets. All net types seemed to be effectively protected against UV-light.

## Background

Malaria is one of the major public health problems for most of the developing countries in the world. The 2012 world malaria report [1] states that around 99 countries still suffer from malaria transmission. In 2010 it was estimated that the disease killed 655 000 people of which 90% in the African Region. In order to control and prevent this disease, governments, researchers and manufactures work together to find the best solution. In 1991–92, the World Health Organization (WHO) organized 3 interregional meetings to develop an updated global strategy to fight malaria. The Ministerial Conference on Malaria adopted its global strategy as well as a World Declaration on the Control of Malaria. The four main components of this strategy are as follows: 1. disease management through early diagnosis and prompt treatment, 2. planning and application of selective and sustainable preventive measures including vector control, 3. early detection or prevention of epidemics and their containment, and 4. regular assessment of the malaria situation [2]. Vector control remains the most generally effective measure to prevent malaria transmission. It is one of the basic technical elements of the Global Malaria Control Strategy [3], [4]. One of the best methods to prevent malaria still is the use of bed nets treated with insecticides [5]–[8].

According to the 2011 world malaria report [9], international funding for malaria control continuously increased up to US$ 2 billion in 2011. The budget for malaria control has enabled endemic countries to greatly increase access to insecticide-treated mosquito nets (ITNs); the percentage of households owning at least one ITN in sub-Saharan Africa is estimated to have risen from 3% in 2000 to 50% in 2011 while the percentage protected by indoor residual spraying (IRS) rose from less than 5% in 2005 to 11% in 2010. Also from the same source [9], household surveys indicate that 96% of persons with access to an ITN within the household actually use it. That confirmed the statement that a bed net in a perfect condition prevents 90% of bites [10], and stays the main route for the vector control. Also, when one ITN is used in a house, it reduces the number of mosquito bites experienced by others sleeping without a net in the same house [11]–[13].

Conventionally treated mosquito nets (ITNs) evolved into a new generation of nets called Long-Lasting Insecticidal Mosquito Nets (LLINs or LNs). This evolution overcomes some problems. Problems like the accuracy of dosage of the formulation under field condition (to get the right concentration over the nets) and also problems like exposure to insecticide during nets re-retreatment [14] are avoided. Also because less insecticides are removed after washing process, the nets remain more efficient [15]. The new nets do not have to be retreated with pesticides after a certain time. Many studies have been reported on this issue [3], [14], [16]–[18]. Most of them only focus on the efficacy and washing resistance using the WHO standards. The common detergent used is “Savon de Marseille”. Even though this soap is not standardized; it is still recommended by WHO for the evaluation of the wash resistance of nets, because it is close to different soaps and detergents used in practice. Many questions still remain open, because of the limited information on the effects of different washing and drying methods [19], [20]. For instance “What happens with the active ingredient when nets are washed in different ways” such as hand washing versus machine washing? During the 54^th^ Collaborative International Pesticides Analytical Council (CIPAC) Technical Meeting in 2010 the question was raised how to standardize the WHO washing or the laboratory hand washing simulation procedure by using a standardized washing agent [21]. No data were available to compare the proposed washing agent called CIPAC washing agent with the commonly used detergent. Today, pyrethroids are the only class of insecticides recommended for the treatment of mosquito nets [22] but it seems that UV light in sunlight has some degrading effects on the insecticide deposit on the nets [18], [23]. Based on this, LN manufacturers advice the customers to dry nets in the shadow without direct sun light. This recommendation is also used in many others studies on efficacy of LNs [24]. Therefore the objectives of the current research were:

- To evaluate the effect of washing on the release of active ingredient content for different brands of nets and for coated versus incorporated technologies.
- To evaluate the effect of UV light on the release of active ingredient content of these nets.
- To compare the laboratory hand washing simulation with the proposed CIPAC detergent to a domestic washing method (ISO 6330∶2000 machine washing for textile testing).

## Materials and Methods

### Materials

Two technologies of mosquito nets were considered in this study.

Coated technology: In this technology a resin based polymer coating is used as the insecticide reservoir for replacement of surface insecticide and this coating is bonded to the surface of the filament [17]. This technology is usually applied to multifilament polyester textile nets. Two types of coated nets were used for this study: Interceptor® nets treated with alpha-cypermethrin (200 mg a.i./m^2^ of net) provided by BASF Chemical Company and PermaNet®2.0 nets treated with deltamethrin (55 mg a.i./m^2^ of net) provided by Vestergaar Frandsen SA.

Incorporated technology: In this technology, the pyrethroid insecticide is directly incorporated into the textile fibers from which the netting is made. This technology can be applied for polyethylene nets [17]. The insecticide diffuses constantly over time to the surface of the yarn and will be regenerated from the reservoir after the surface insecticide is washed off or is lost otherwise. The bioavailability of the active ingredient is designed to be sufficient to kill the mosquitoes [25]. One type of incorporated nets is used in this study: Netprotect® nets. These nets are treated with deltamethrin (79 mg a.i./m^2^ of net) and provided by the Dean Superior Textile Co.

### Preparation of Net Samples

For the laboratory hand washing simulation and the indoor drying, each type of net was cut into 63 pieces of 25 cm×25 cm. For the ISO 6330∶2000 machine washing and the indoor and UV drying, 20 bigger pieces of about 70 cm×70 cm were cut from each type of net.

## Methods

### Washing Procedure

Two washing procedures were tested to know their impact on active ingredient content of the nets.

Method-1 corresponds to the WHO washing procedure [26], except that the CIPAC standard washing agent is used instead of “Savon de Marseille”. It is further on referred to as laboratory hand washing simulation.

Method-2 is the ISO 6330∶2000 domestic washing further on referred to as the ISO 6330∶2000 machine washing. It is considered to be the more stringent [27] European standard for domestic washing of textiles.

Both methods are standardized. Therefore, it was possible to compare with other studies using these washing procedures.

#### Method-1: Laboratory hand washing simulation

Preparation of CIPAC washing agent according to Yumiko Kozuki and Tsunehisa Fujita [21], [28]: A bottle containing polyoxyethylene glycol (25) monostearate was heated up to 50°C in order to decrease its viscosity. Then, 12.0 g of sodium oleate and 8.0 g of polyoxyethylene glycol (25) monostearate were successively weighed in the same flask containing 80 mL of deionised water at room-temperature. The mixture was stirred and heated up to 50°C until it became clear and homogeneous. This CIPAC washing agent was kept for 4 weeks. The flask was stored in a dark cool place.

Polyoxyethylene glycol (25) monostearate and sodium oleate (≥82% fatty acids) were both supplied by Sigma-Aldrich.

Washing process: In a 1 liter glass bottle, 4.0 g±0.1 g of CIPAC washing agent (polyoxyethylene glycol (25) monostearate+sodium oleate+water) was dissolved in 500 mL of deionised water at 30°C±3°C. The pH of the solution with the detergent was 9.45. Net samples (25 cm×25 cm) were individually put into the bottle and washed by shaking for 10 minutes in a horizontal shaker (type SM 30 B Control provided by Edmund Bühler GmbH company) set at 155 beats per minute (rpm) with an amplitude of 15 mm. Then, samples were removed and rinsed twice for 10 minutes in clean deionised water at 30°C±3°C in the same shaking conditions as stated above. The average temperature during the washing process was 30.4°C with a standard deviation of 0.6 (n = 57).

The nets were dried with the indoor drying procedure, as described below. The next washing was done seven days after the previous washing in order to take into account the regeneration time of the net. During this period, the nets were stored in a room at 30°C, which is considered as a reasonable regeneration temperature [29]. Then, three pieces of 25 cm×25 cm were randomly removed and stored into a freezer for assuring the preservation and for determination of the active ingredient content in the nets by chromatographic analysis. In total up to 20 washes were performed.

#### Method-2: ISO 6330∶2000 machine washing

This method was carried out in the Department of Textiles of Ghent University. The net samples were placed in the automatic washing machine type A - horizontal axis front loading type. Sufficient ballasts of 100% polyester were added to reach a machine load of 2 kg. 20 g of IEC A* detergent (non-phosphate reference detergent) was added into the dispenser of the machine. A gentle washing program was performed. There was no agitation during the heating up to the set temperature ±5°C [30]. The set temperature was 30°C. The hardness of the water used was less than 2 dh. After the washing cycle was completed, the net samples were dried according to one of the drying procedures described below. All the samples were washed at 7-days intervals up to 20 washes in order to take into account the regeneration time of the net. During regeneration time, net samples were stored in a room at 30°C which is a reasonable regeneration temperature [29], and then, three (3) pieces of 25 cm×25 cm were randomly cut from the concerned group of net samples and kept into a freezer for assuring the preservation and for determination of the active ingredient content in the nets by chromatographic analysis. The IEC A* detergent was provided by SDL International company.

### Drying Procedure

Two drying procedures were tested to study their impact on active ingredient content of the nets.

#### Method A: Simulation of indoor drying

**Figure 1:**
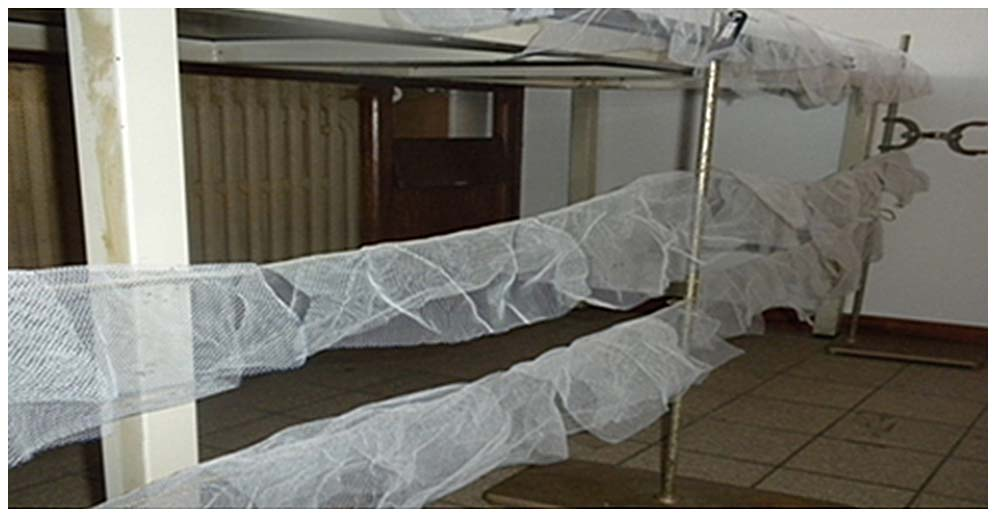
Samples for indoor drying. The samples were hung on the cords which were horizontally attached on fixed supports in the room. The position of the curtains was lowered during the drying time to avoid direct sunlight.

The net samples washed with method 1 and one group of net samples washed with method 2 were hung on a line in a room without sunlight as shown in the Figure 1 for 24 hours. The average temperature inside the room was 24°C.

#### Method B: Simulation of outdoor drying with UV light

The second group of net samples washed according to method 2 was dried by exposure during 24 hours to a “True-Light” lamp. The lamp was provided by True-Light International GmbH Company. The spectrum of this lamp has been developed to simulate daylight for indoor environments. Its main characteristics are: rated luminous flux at 25°C = 1200 lm, correlated color temperature = 5500 K, part of UVA in the spectrum 3.0%, part of UVB in the spectrum 0.3%. (remark: UVA radiation is about 95% of the solar UV light reaching the earth surface [31]. UVA and UVB wavelengths are respectively ranged as 315 nm –400 nm and 280 nm –315 nm [32]).

**Figure 2:**
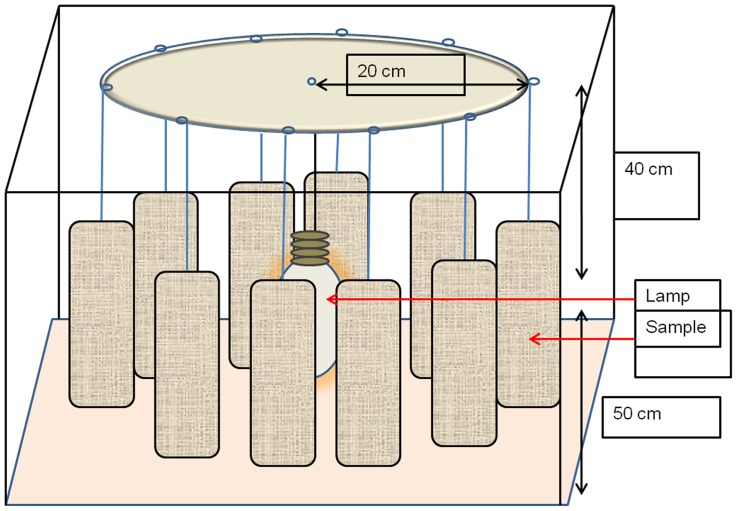
Samples for outdoor drying with UV light. The samples were hung on cords which were tied around the circumference of a circular plate. The True-Light lamp was fixed at the center. In this way the distance between all nets and the light was equal.

The net samples were hung as shown in the Figure 2. The temperature in the drying room was 22°C on average. This value is below that used in some studies to accelerate the insecticide migration on the surface of the net. Because the temperature is kept low, heat is not taken into account in this UV impact study.

### Chemical Analysis

Because it was expected that the samples would have low amounts of active ingredient due to the impact of the washing cycles, a multi-residue method able to detect deltamethrin and alpha-cypermethrin in coated and incorporated nets with a high sensitivity was used [33]. The analyses were done at the laboratory of Crop Protection Chemistry of the Ghent University. 300 mg (to the nearest 0.1 mg) of sample was weighed into a 100 mL conical flask. The active ingredient was extracted by heating under reflux during 30 minutes with 40 mL of xylene. The extract solution was allowed to cool down to the ambient temperature and was filtered through a büchner funnel using Whatman™ filter paper into a 50 mL volumetric flask. The filtration cake was rinsed and the extract solution was extended to 50 mL with xylene. After that, 1 mL of the extract solution was diluted into 10 mL with xylene and a portion of this solution was transferred into a vial for chromatographic injections.

The extract was analyzed by gas chromatography with electron capture detection (GC-µECD) using an Agilent Technologies 6890 N equipped with an auto sampler Agilent Technologies 7683 Series injector which was used in split mode. The chromatographic separation was performed with a HP-5 (5% Phenyl Methyl Siloxane) capillary column (30 m×0.250 mm i.d., 0.25 µm film thickness). Helium was used as the carrier gas and kept at constant pressure of 102.7 kPa with a nominal minimal flow of 0.9 mL/min. The split ratio, split flow and total flow were respectively 50∶1, 45.5 mL/min and 49.9 mL/min. The µECD detector temperature was 300°C with nitrogen as make-up gas kept at a constant flow of 60.0 mL/min. For each sample two chromatographic injections were done and the mean was reported as mass of active ingredient per unit mass of netting (g/kg), by comparison with an external standard calibration curve. Then, the value was converted into mg/m^2^ according to the given density of the net. The injection volume was 1 µL and the oven temperature was programmed as: isothermal at 130°C for 1 min, from 130°C to 280°C at 30°C/min and held for 16 min.

### Characterization of Washing Resistance: Measurement of Retention/Release and Surface Concentration of Active Ingredient

According to A. Hill in the report of the 11^th^ WHOPES Working Group Meeting [34], WHO standard bioassays cannot be used throughout the world for quality control purposes. So, physico-chemical tests must be used. But the use of such tests to define efficacy is problematic. As a minimum for the development of a meaningful specification, it is essential to have a detailed knowledge of the release/retention characteristics of the product. Data were computed to check firstly, if the main characteristic of the LNs could be related to the number of washing cycles. Secondly it was checked which trend or mathematic model could be used to fit the relationship between the release of the active ingredient from the LNs and the number of washing cycles. The data were fitted with the curve estimation program from SPSS 20 and 4 mathematical models (linear, logarithmic, quadratic and exponential) were tested. To check whether the main characteristics of the LNs could be related to the number of washing cycle, the active ingredient retention at each wash was calculated as:
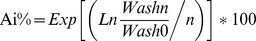
with wash_n_ the active ingredient content after the n wash and Wash_0_ the initial active ingredient content of the unwashed net.

### Effect of Washing Method (Comparison of Laboratory Hand Washing Simulation versus ISO 6330∶2000 Machine Washing)

Two groups of samples were considered from each type of LN. The first consisted of 10 pieces of about 70 cm×70 cm. This group was washed following the ISO 6330∶2000 machine washing procedure (method-2) up to 20 times. The second group consisting of 60 pieces of 25 cm×25 cm was washed following the laboratory hand washing simulation procedure (method-1) up to 20 times. Between the washes, both groups were dried indoor and stored at the same condition before the next wash. To check whether the two washing procedures were significantly different, the active ingredient content of the 1, 5, 11, 15 and 20 times washed samples for both groups was analyzed by GC. Data obtained were analyzed with two-way ANOVA using the washing procedure as fixed factor with 2 levels (laboratory hand simulation and ISO 6330∶2000 machine washing) and the number of washes as fixed factor with 5 levels.

### Effect of Drying: Indoor Drying versus Outdoor Drying with UV Light

The effect of 2 drying methods was compared on all types of nets washed according to method-2.

To assess the effect of the drying process on the release of the active ingredient from the nets, two groups of samples were considered from each type of LN. Each group contained 10 samples of about 70 cm×70 cm washed following the ISO 6330∶2000 machine washing procedure up to 20 times. After each wash, one group of samples was dried during 24 hours indoors as shown in Figure 1. The other group was dried during 24 hours with true-light lamps as shown in Figure 2.

To check whether the drying process had a significant impact on the active ingredient content of LNs, the samples from each group were subjected to GC analysis.

### Statistics

The software SPSS version 20 package was used to run all the statistic tests. Statistical analysis was performed using Student’s t-test and ANOVA. Before performing ANOVA and using obtained data, assumptions (homogeneity of variance, normality of observations) underlying the analysis of variance were tested. Comparisons were made between the number of washes, the type of washing process, the type of drying methods and the type of impregnation technology. To find the best relationship between the release of active ingredient from the LN and the wash numbers the curve estimation program was used.

## Results and Discussions

### Active Ingredient Content in LN after Different Washing Cycles

Table 1 shows the average of active ingredient content in LN after different washing cycles. Many studies confirmed that the washing process affects significantly the release of active ingredient content from the nets [12], [34], [35]. Here, independently to the drying process, it was checked whether the laboratory hand washing simulation using the CIPAC washing agent and the ISO 6330∶2000 machine washing as described above affected significantly the total active ingredient content on the different nets. Data obtained are shown in Table 2.

**Table 1:** Active ingredient content in LN after the washing cycles according to the number of washes.

Cycle	N° ofwash	Range ofwashes	Interceptor®			PermaNet®2.0			Netprotect®		
			g a.i./kgof net	mg a.i./m^2^of net	Insecticideretention (%)	g a.i./kgof net	mg a.i./m^2^of net	Insecticideretention (%)	g a.i./kgof net	mg a.i./m^2^of net	Insecticideretention (%)
**Laboratory hand washing simulation** **– Indoor drying**	0	0–5	4.72	188.89	68.7	2.00	59.86	52.1	1.12	49.38	78.0
	1		3.70	147.97		1.59	47.70		1.04	45.77	
	3		3.35	133.90		1.26	37.75		0.97	42.50	
	5		3.24	129.68		1.04	37.21		0.88	38.52	
	7		3.32	132.75		0.81	24.36		1.04	45.81	
	9		2.25	90.08		0.61	18.41		0.88	38.60	
	11		1.91	76.38		0.69	20.75		0.99	43.67	
	13		1.60	63.96		0.70	20.87		0.99	43.45	
	15	15–20	1.52	60.70	68.1	0.65	19.44	78.4	0.86	37.65	83.8
	17		1.17	46.79		0.48	14.38		0.84	37.01	
	19		1.19	47.55		0.52	15.58		0.80	35.14	
	20		1.03	41.36		0.51	15.25		0.72	31.55	
**ISO 6330∶2000 machine washing – Indoor drying**	0	0–5	4.72	188.89	56.5	2.00	59.86	43.7	1.12	49.38	94.8
	1		3.64	145.42		1.51	45.44		1.06	46.80	
	3		3.43	137.19		1.09	32.72		0.85	37.38	
	5		2.67	106.81		0.87	26.14		1.06	46.79	
	7		4.47	98.87		0.71	21.35		1.06	46.54	
	9		2.73	109.00		0.58	17.42		1.03	45.29	
	11		1.96	78.57		0.65	19.63		1.06	46.57	
	13		1.99	79.41		0.57	17.02		0.79	34.96	
	15	15–20	1.68	67.22	92.4	0.50	14.98	105.2	0.99	43.35	79.5
	17		1.65	66.02		0.51	15.25		0.80	35.36	
	19		1.38	55.05		0.41	12.37		0.86	37.99	
	20		1.55	62.10		0.53	15.76		0.78	34.46	
**ISO 6330∶2000 machine washing – UV drying**	0	0–5	4.72	188.89	56.3	2.00	59.86	55.1	1.12	49.38	92.9
	1		4.12	164.95		1.48	44.53		1.09	48.03	
	3		3.51	140.51		1.23	36.83		0.99	43.48	
	5		2.66	106.38		1.10	32.97		1.04	45.87	
	7		2.32	92.97		0.75	22.43		1.08	47.64	
	9		2.48	99.02		0.71	21.29		1.10	48.47	
	11		2.40	96.11		0.67	20.22		0.95	41.78	
	13		2.11	84.35		0.61	18.24		0.95	41.84	
	15	15–20	2.06	82.60	71.9	0.59	17.59	98.2	0.94	41.25	84.4
	17		1.55	61.86		0.60	18.05		0.85	37.23	
	19		1.87	74.89		0.44	13.21		0.83	36.34	
	20		1.49	54.41		0.58	17.27		0.79	34.81	

The washes were done up to 20 times. 3 samples (repetitions) after each washing cycle were analyzed. The average insecticide content of the samples from each odd number of washings was recorded and also the percentage of the active ingredient retention was calculated after each 5 washing.

**Table 2:** Summary of statistic tests of the effect of washing on LN (One way ANOVA).

Cycle (Washing/drying)	Nets	Test	Chi-Square or F	Degree of freedom		P-value
				BetweenGroup	WithinGroup	
**Laboratory hand washing simulation/indoor drying**	**Interceptor®**	Kruskal Wallis	33.738	12		0.001
	**PermaNet®2.0**	Kruskal Wallis	27.037	10		0.003
	**Netprotect®**	Single factor ANOVA	5.902	10	22	0.000
**ISO 6330∶2000 machine washing/indoor drying**	**Interceptor®**	Single factor ANOVA	6.983	10	22	0.000
	**PermaNet®2.0**	Single factor ANOVA	20.382	10	22	0.000
	**Netprotect®**	Single factor ANOVA	10.910	10	22	0.000
**ISO 6330∶2000 machine washing/UV drying**	**Interceptor®**	Single factor ANOVA	5.005	10	22	0.001
	**PermaNet®2.0**	Kruskal Wallis	25.947	10		0.004
	**Netprotect®**	Single factor ANOVA	7.833	10	22	0.000

The assumptions underlying the analyses of variance were satisfied. The type and number of washes had a significant effect on the loss of active ingredient content.

The Table 2 shows that there is a very significant difference in the concentration of the active ingredient between the washing cycles of the coated nets (Interceptor® and PermaNet®2.0) (0.001<p<0.01). This difference is very highly significant for the incorporated nets (Netprotect®) (p<0.001). The type of washing and the number of washing cycles both have effect on the release of the active ingredient content of the net.

### Characterization of Wash Resistance: Measurement of Retention/Release and Surface Concentration of Active Ingredient Retention/Release of Active Ingredient from LNs

Chemical analyses showed that the initial active ingredient concentration from Interceptor® and PermaNet®2.0 nets were respectively 4.72 g/kg (189 mg/m^2^) and 2.00 g/kg (60 mg/m^2^). These concentrations were slightly different compared to the target dose of 5 g/kg (200 mg/m^2^) for Interceptor® nets and 1.83 g/kg (55 mg/m^2^) for PermaNet®2.0 nets but within the specifications of ±25% [36]. The analysis of Netprotect® nets showed 2 peaks for deltamethrin with a proportion of about 26/74. The first eluting peak was the *R*-alpha isomer of deltamethrin, a non relevant impurity of deltamethrin. The initial deltamethrin concentration of Netprotect® was 1.12 g/kg.

The Table 1 shows that the retention of ≥50% of the active ingredient after 5 standard washes according to each type of net is met [34]. That would indicate a retention index of ≥90% for reservoir behavior or ≥87% for free migration behavior. This was confirmed in Table 3, where the active ingredient retention at each wash was calculated.

**Table 3:** Estimated insecticide retention per wash.

Type of net		Estimated variation of insecticide retention per wash (%)			
		Laboratory hand washing simulation/Indoor drying	ISO 6330∶2000 machine washing/Indoor drying	ISO 6330∶2000 machine washing/UV drying	Literature
**Interceptor®**	Range	78.3–95.1	77.0–94.6	87.3–95.2	
	Average with 95% CI	91.1 [88.1, 94.1]	91.2 [87.8, 94.6]	92.4 [90.7, 94.2]	
**PermaNet®2.0**	Range	79.7–93.4	75.9–93.5	74.4–94.0	
	Average with 95% CI	89.4 [86.6, 92.2]	87.8 [84.2, 91.4]	88.9 [85.2, 92.6]	
**Netprotect®**	Range	92.7–99.0	91.1–99.5	95.8–99.8	98.7–101.7 [34]
	Average with 95% CI	97.2 [95.9, 98.6]	97.6 [95.9, 99.3]	98.4 [97.6, 99.1]	99,7 [34] 98.8 [38]

For each type of LN, the average insecticide retention per wash and the 95% confidence interval is calculated.

**Figure 3:**
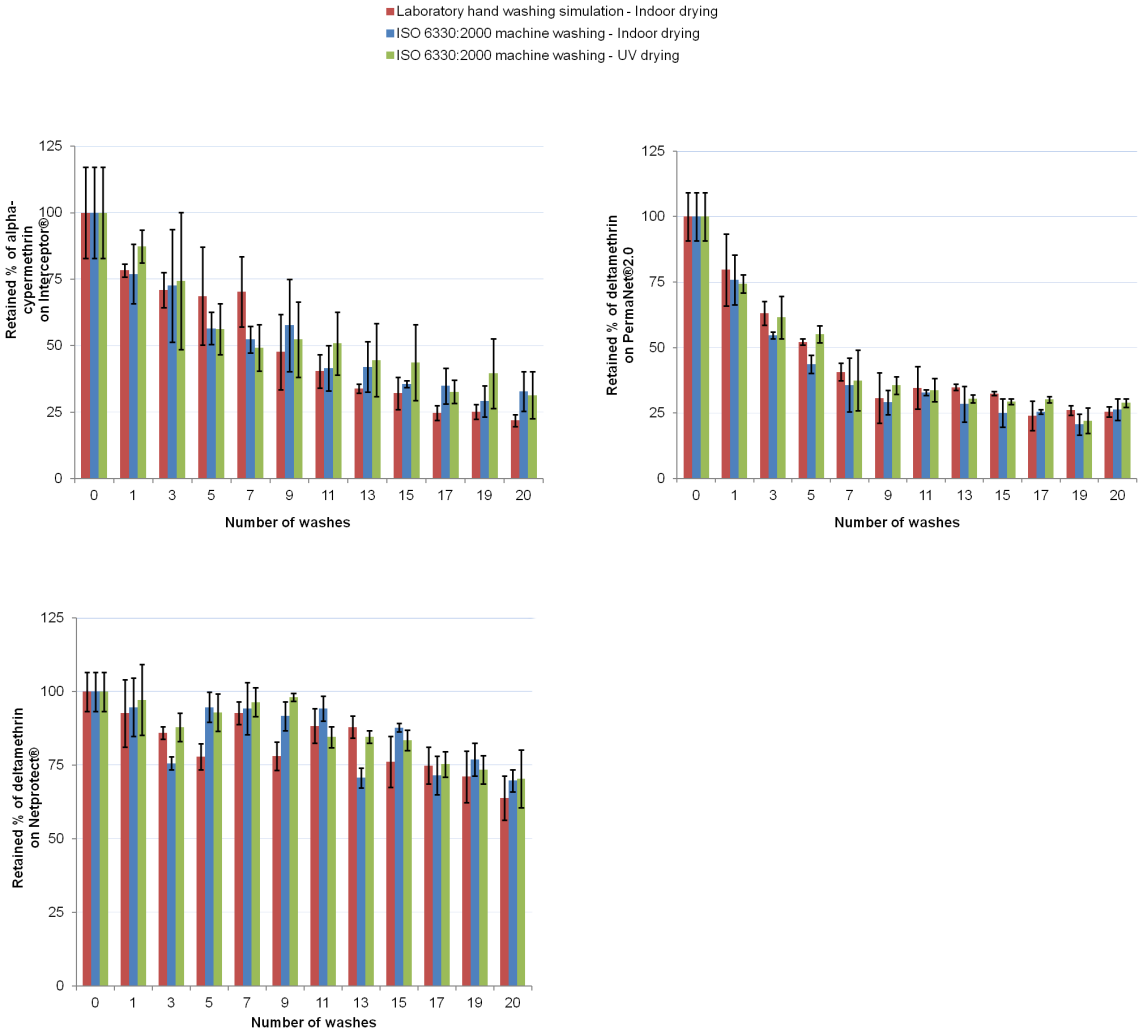
Percentage of residual insecticide on nets. **A.** The Interceptor® nets lose active ingredient after each wash, independent of the type of washing or drying. Only around 29% of the baseline concentration of alpha-cypermethrin stayed on the nets after 20 washes. **B.** PermaNet®2.0 nets lose active ingredient after each wash, independent of the type of washing or drying. Only around 27% of the baseline concentration of deltamethrin stayed on the nets after 20 washes. **C.** Netprotect® nets lose active ingredient after each wash, independent of the type of washing or drying. The remaining insecticide after 20 washes is about 70% of the baseline concentration.

From Figure 3, it can be noticed that the percentage of alpha-cypermethrin remaining on Interceptor® LN after 20 washes cycles was 21.9% (1.03 g a.i./kg, n = 3) for the laboratory hand washing simulation (indoor drying), 32.9% (1.55 g a.i./kg, n = 3) for the ISO 6330∶2000 machine washing (indoor drying) and 31.5% (1.49 g a.i./kg, n = 3) for the ISO 6330∶2000 machine washing (UV drying).

The overall percentage of alpha-cypermethrin remaining on Interceptor® LN after 20 wash cycles was 28.7% (95% CI [22.7, 34.8]).

From the Figure 3, it can be noticed that for the PermaNet®2.0 LN, deltamethrin remaining on the net was 25.5% (0.51 g a.i./kg, n = 3) for the laboratory hand washing simulation (indoor drying), 26.3% (0.53 g a.i./kg, n = 3) for the ISO 6330∶2000 machine washing (indoor drying) and 28.9% (0.58 g a.i./kg, n = 3) for the ISO 6330∶2000 machine washing (UV drying).

The overall percentage of deltamethrin remaining on PermaNet®2.0 LN after 20 washes was 26.9% (95% CI [24.7, 29.1]). These data confirmed those of the study of Atieli [19] which showed that PernaNet®2.0 retained about 18 to 27% of the deltamethrin after 20 washes. Using standardized WHO protocol washing [26], the study of V. Corbel [37] showed also that PermaNet®2.0 loses between 60 to 85% of deltamethrin after 20 washes.

From the Figure 3, it can be noticed that for the Netprotect® LN, the percentage of deltamethrin remaining in the net after 20 washes cycles was 63.9% (0.72 g a.i./kg, n = 3) for the laboratory hand washing simulation (indoor drying), 69.8% (0.78 g a.i./kg, n = 3) for the ISO 6330∶2000 machine washing (indoor drying) and 70.5% (0.79 g a.i./kg, n = 3) for the ISO 6330∶2000 machine washing (UV drying).

The overall percentage of deltamethrin remaining in Netprotect® LN after 20 washes was 68.0% (95% CI [62.5, 73.6]). This was also shown by Skovman *et al.* [38] (77% of deltamethrin remained on Netprotect® after 20 washes) and in the 11th WHOPES report [34] (77.6% remains). The high percentage of deltamethrin retention for Netprotect® is due to the incorporation technology compared to the coated one. This may also mean that the active ingredient from the Netprotect® net is more inaccessible to aqueous surfactant and is also unlikely to be accessible for mosquitoes walking on the surface [34]. So, from our observation and based on this fact, Netprotect® net might perform less against mosquitoes compared to PermaNet®2.0 and Interceptor®. This assertion has to be confirmed by bioassay.

### Efficacy of Nets after Washing Process (Wash Resistance and Efficacy of the Nets)

As this study did not involve bioassay tests, the data were compared with data from the literature on active ingredient content on the net (mg/m^2^) with the corresponding mortality percentage according to the number of washes. To assess the efficacy of mosquito nets, bioassays are recommended by WHOPES, even if results in different parts of the world are varying. A net maintains its efficacy if it produces more than 80% mosquitoes mortality in a bioassay cone test after 20 washes [26]. The variation was in general due to the formulation of the insecticide, the type of insecticide, the mosquito species, the susceptibility level of mosquitoes, the time of exposure, the texture of bed net, the quality of different batches and the type of tests. Some studies revealed that there was no significant difference in the mortality of species exposed to different types of LNs washed by machine or by hand [10], [16], [25], [39]. Table 4 compares information about the efficacy of the nets submitted to the 3 wash cycles used during this study. It shows that the active ingredient content is close to values published in the literature studies until 20 washes for all the 3 types of nets tested. Indeed, the PermaNet®2.0 nets after 20 washes should provide 70% mortality [19] with around 15 mg a.i/m^2^, while the Netprotect® and Interceptor® nets should provide respectively a mortality of 30% [19] with 30 mg a.i/m^2^ and 15% [10] with around 60 mg a.i/m^2^. Thus, even if the high percent of retention seems to be less favorable for Netprotect® nets in term of availability of the insecticide over the net, the Table 4 infirm this trend.

**Table 4:** Comparison of total active ingredient content (in mg/m^2^) and literature bioassay data.

	N° of wash	Mean (mg a.i./m^2^ with 95% CI) during the processes			Data from the literature about active ingredient content on net (mg/m^2^) with mortality percentage	
		Laboratory wash - Indoor drying	ISO 6330∶2000 wash - Indoor drying	ISO 6330∶2000 wash –UV drying	mg a.i/m^2^ [authors]	Mortality of conical test (%)/species
**Interceptor®**	0	188.9 [108.2, 269.6]	188.9 [108.2, 269.6]	188.9 [108.2, 269.6]	211.1±27.2 [10]	100.0/An.stephensis
					200 [19]	100.0/An. gambiae
	1	148.0 [136.7, 159.2]	145.4 [93.2, 197.7]	165.0 [135.8, 194.1]	191.1±12.4 [10]	100.0/An.stephensis
	2				167.8±13.3 [10]	100.0
	3	133.9 [103.0, 164.8]	137.2 [37.8, 236.6]	140.5 [19.5, 261.5]	140.5±23.1 [10]	100.0/An.stephensis
	5	129.7 [43.3, 216.0]	106.8 [78.2, 135.4]	106.4 [61.6, 151.2]	99.6–160 [19]	50–75/An. gambiae
	6				118.6±19.1 [10]	97.5±1.0/An.stephensis
	8				115.2±8.9 [10]	88.8±2.2
	10				95.8–120 [19]	35–65/An. gambiae
	15	60.7 [32.0, 89.4]	67.2 [61.1, 73.3]	82.6 [15.7, 149.5]	90.6±8.4 [10]	72.5±3.2/An.stephensis
					83.6–112 [19]	20–50/An. gambiae
	20	41.4 [30.8, 51.9]	62.1 [26.9, 97.3]	59.4 [18.1, 100.7]	61.2±2.8 [10]	15.0±2.5/An.stephensis
					81–109 [19]	20–40/An. gambiae
**PermaNet®2.0**	0	59.9 [46.2, 73.5]	59.9 [46.2, 73.5]	59.9 [46.2, 73.5]	55 [19]	100/An. gambiae
					66.7 [50]	100/An. gambiae
	1	47.7 [27.3, 68.1]	45.4 [31.3, 59.6]	44.5 [39.4, 49.7]		
	3	37.8 [31.0, 44.5]	32.7 [30.7, 34.7]	36.8 [24.7, 49.0]		
	5	31.2 [29.4, 33.1]	26.1 [21.1, 31.2]	33.0 [28.1, 37.8]	18.98–30.03 [19]	83–93/An. gambiae
					53.36 [50]	100/An. gambiae
	10				15.015–22.825 [19]	78–90/An. gambiae
					45.356 [50]	
	15	19.4 [18.4, 20.5]	15.0 [6.9, 23.0]	17.6 [16.1, 19.1]	11.99–16.61 [19]	43–85/An. gambiae
	20	15.3 [12.3, 18.2]	15.8 [9.6, 21.9]	17.3 [14.8, 19.7]	10.01–15.125 [19]	28–70/An. gambiae
					27.347 [50]	100/An. gambiae
**Netprotect®**	0	49.4 [41.3, 57.5]	49.4 [41.3, 57.5]	49.4 [41.3, 57.5]	65 [19]	100/An. gambiae
					1.95 g/kg [34]	100/An. gambiae
	1	45.8 [31.7, 59.8]	46.8 [34.7, 58.9]	48.0 [33.3, 62.8]		
	3	42.5 [39.8, 45.2]	37.4 [34.6, 40.1]	43.5 [37.6, 49.4]		
	5	38.5 [33.1, 43.9]	46.8 [40.5, 53.1]	45.9 [38.1, 53.7]	35.815–45.045 [19]	45–58/An. gambiae
					[34]	97/An. gambiae
	10				26–41.6 [19]	35–50/An. gambiae
					[34]	99/An. gambiae
	15	37.7 [27.1, 48.2]	43.4 [41.6, 45.1]	41.3 [36.9, 45.6]	25.415–34.385 [19]	23–34/An. gambiae
					[34]	78/An. gambiae
	20	31.5 [22.3, 40.8]	34.5 [29.8, 39.1]	34.8 [22.7, 46.9]	22.425–30.225 [19]	15–28/An. gambiae
					1.52 g/kg [34]	76/An. gambiae

The results obtained in this study and the literature data about the efficacy of the same nets are put next to each other. It shows that the active ingredient content is close to values published in the literature for all the types of the nets tested.

### Effect of the Wash Cycles and Fitting Curve (Mathematical Models of Active Ingredient Retention in Function of the Washing)

**Figure 4:**
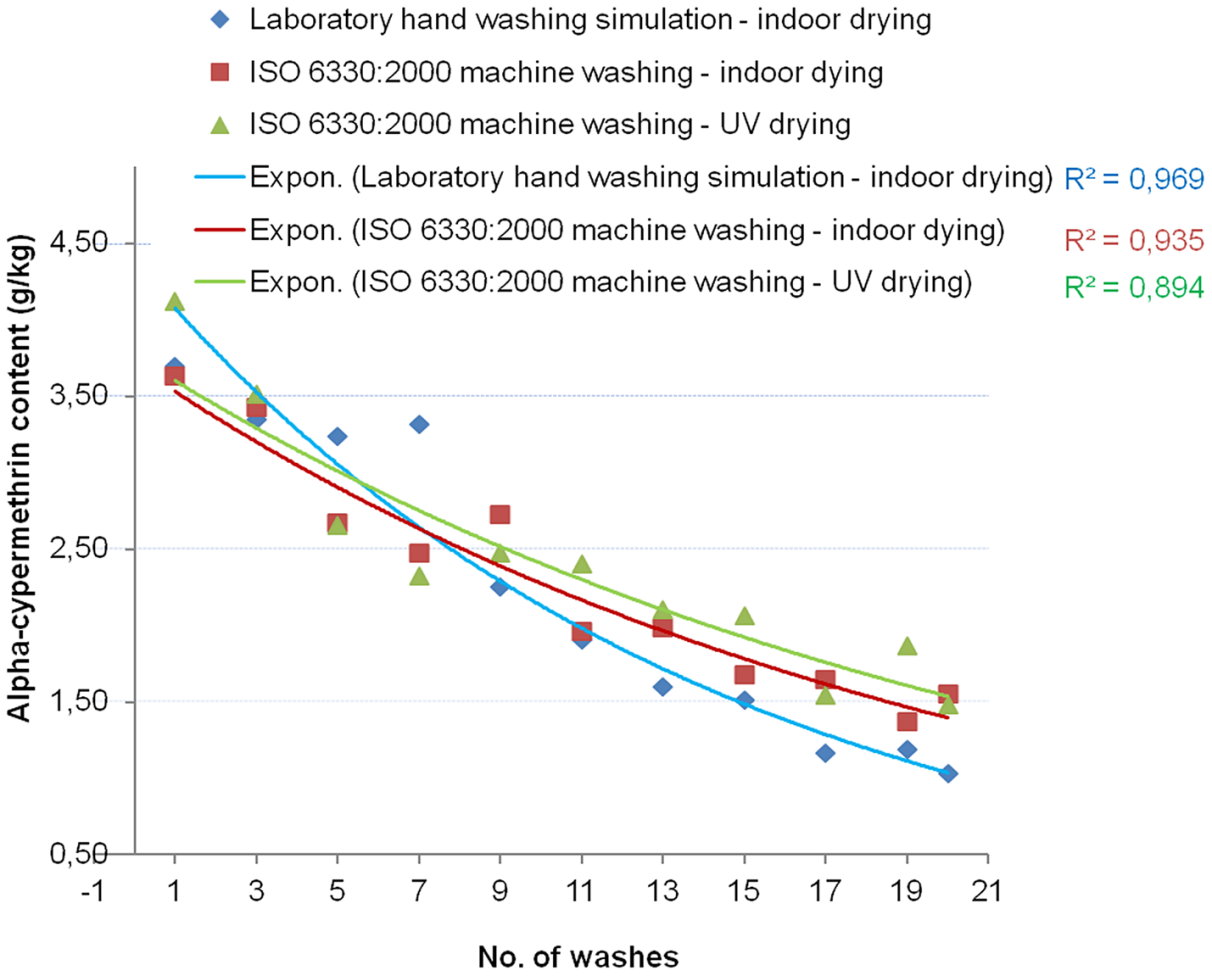
Effect of washing on alpha-cypermethrin total content of Interceptor®: exponential curves fitted to the data. The exponential curve fitting of the data for each of the 3 washing cycles is shown. The concentration of the insecticide on the net as an exponential function of the number of washes is then shown.

**Figure 5:**
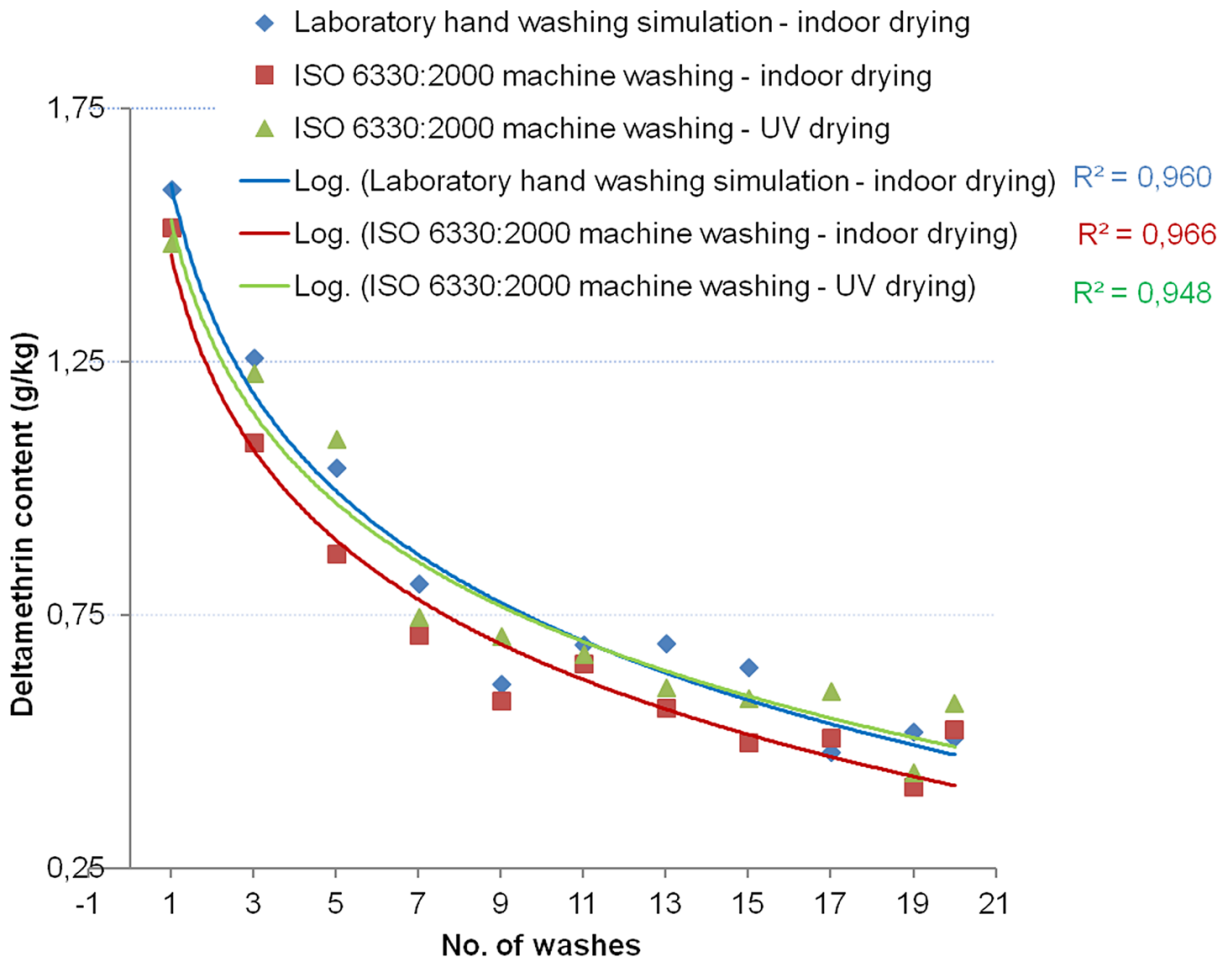
Effect of washing on deltamethrin total content of PermaNet®2.0: logarithmic curves fitted to the data. The figure shows the logarithmic curve fitting the data for each of the 3 washing cycles performed. The concentration of the insecticide on the net as a logarithmic function of the number of washes is then shown.

Table 5 summarizes the F test of model fit and the estimated parameters of the models. The significant value of the F statistics on each brand of LN is less than 0.05 for the 4 models (linear, logarithmic, quadratic and exponential). This means that the variation explained by each model was not due to the chance. R, the multiple correlation coefficient, is the linear correlation between the observed and model-predicted values of the insecticide content. Its large value indicates a strong relationship. That was also seen by the R-Square. R-Square statistic, a measure of the strength of association between the observed and model-predicted values of the dependent variable (concentration of active ingredient of the nets), was large in general for all the nets, except for the modeling of the Netprotect® nets. Indeed for Netprotect®, the washing cycle’s data did not properly fit any curve model. For the others types of net the large R-Square values indicated strong relationships for exponential, logarithmic and quadratic models. A comparison between the 3 cycles showed that the R-Square for the logarithmic model were larger for PermaNet®2.0 nets while, for the Interceptor® nets it appeared that the exponential model better follows the trend of the data (Figure 4 and 5). This is confirmed by the findings of A. Hill [34].

**Table 5:** Models Summary and Parameter Estimates.

Type of nets	Cycles	Equation^b^		F-test of the model					Model parameters		
			R	R- Square	F	df1	df2	Sig.	Constant(b0)	b1	b2
**Interceptor®**	ISO 6330∶2000 wash –Indoor drying	Linear	0.832	0.692	69.794	1	31	0.000	3.511	− 0.112	
		Logarithmic	0.828	0.685	67.421	1	31	0.000	3.96	− 0.790	
		Quadratic	0.848	0.720	38.509	2	30	0.000	3.836	− 0.203	0.004
		**Exponential**	**0.864**	**0.746** a	91.073	1	30	0.000	3.656	− 0.049	
	ISO 6330∶2000 wash –UV drying	Linear	0.760	0.577	42.279	1	31	0.000	3.635	− 0.112	
		**Logarithmic**	**0.808**	**0.653** a	58.455	1	31	0.000	4.197	− 0.841	
		Quadratic	0.793	0.629	25.409	2	30	0.000	4.123	− 0.248	0.006
		Exponential	0.772	0.596	45.822	1	31	0.000	3.719	− 0.046	
	Laboratory standardwash – Indoor drying	Linear	0.914	0.835	157.326	1	31	0.000	3.858	− 0.151	
		Logarithmic	0.854	0.729	83.484	1	31	0.000	4.325	−1.000	
		Quadratic	0.918	0.843	80.322	2	30	0.000	4.063	− 0.209	0.003
		**Exponential**	**0.939**	**0.881** a	229.786	1	31	0.000	4.321	− 0.072	
**PermaNet®** **2.0**	ISO 6330∶2000 wash –Indoor drying	Linear	0.824	0.679	65.636	1	31	0.000	1.193	− 0.043	
		**Logarithmic**	**0.943**	**0.890** a	249.909	1	31	0.000	1.462	− 0.350	
		Quadratic	0.930	0.865	96.427	2	30	0.000	1.522	− 0.135	0.004
		Exponential	0.851	0.723	81.078	1	31	0.000	1.201	− 0.054	
	ISO 6330∶2000 wash –UV drying	Linear	0.866	0.749	92.642	1	31	0.000	1.288	− 0.045	
		Logarithmic	0.941	0.885	238.592	1	31	0.000	1.531	− 0.347	
		**Quadratic**	**0.946**	**0.895** a	128.046	2	30	0.000	1.579	− 0.126	0.004
		Exponential	0.873	0.762	99.011	1	31	0.000	1.317	− 0.053	
	Laboratory standardwash – Indoor drying	Linear	0.851	0.724	81.168	1	31	0.000	1.331	− 0.048	
		**Logarithmic**	**0.938**	**0.880** a	227.958	1	31	0.000	1.602	− 0.376	
		Quadratic	0.930	0.864	95.524	2	30	0.000	1.641	− 0.135	0.004
		Exponential	0.873	0.762	99.303	1	31	0.000	1.36	− 0.056	
**Netprotect®**	ISO 6330∶2000 wash –Indoor drying	Linear	0.563	0.317	14.408	1	31	0.001	1.067	− 0.012	
		Logarithmic	0.451	0.203	7.907	1	31	0.008	1.079	− 0.065	
		**Quadratic**	**0.625**	**0.391** a	9.611	2	30	0.001	0.986	0.011	− 0.001
		Exponential	0.565	0.319	14.521	1	31	0.001	1.068	− 0.012	
	ISO 6330∶2000 wash –UV drying	Linear	0.770	0.592	45.036	1	31	0.000	1.125	− 0.015	
		Logarithmic	0.633	0.401	20.753	1	31	0.000	1.146	− 0.086	
		**Quadratic**	**0.817**	**0.667** a	30.010	2	30	0.000	1.049	0.007	− 0.001
		Exponential	0.772	0.596	45.742	1	31	0.000	1.135	− 0.016	
	Laboratory standardwash – Indoor drying	Linear	0.641	0.411	21.662	1	31	0.000	1.041	− 0.012	
		Logarithmic	0.556	0.309	13.85	1	31	0.001	1.066	− 0.074	
		**Quadratic**	**0.690**	**0.476** a	13.615	2	30	0.000	0.971	0.007	− 0.001
		Exponential	0.643	0.413	21.852	1	31	0.000	1.048	− 0.014	

For each type of LN and following the washing cycle, proposed mathematic models with the values of the model’s parameters are given.

a Cycle which fit the model with the largest R-square value compared to the other cycles.

b Linear. Model whose equation is Y = b0+ (b1 * t).

Logarithmic. Model whose equation is Y = b0+ (b1 * ln(t)).

Quadratic. Model whose equation is Y = b0+ (b1 * t)+(b2 * t**2).

Exponential. Model whose equation is Y = b0 * (e**(b1 * t)) or ln(Y) = ln(b0)+(b1 * t).

With t is the number of washes and Y the active ingredient content.

### Effect of Washing Procedure (Comparison of Laboratory Hand Washing Simulation versus ISO 6330∶2000 Machine Washing)

A literature search was done to find out some data coming from Interceptor®, PermaNet®2.0 and Netprotect® nets washed following the laboratory hand washing simulation and using the previous detergent “Savon de Marseille” which had to be replaced. Table 6 presents a comparison between literature data and data obtained in this study. Both data show that the percentage of the insecticide decreases after washing, and in function of the number of washings. When the results obtained in this study are compared to those of the literature, small to big differences are found. Explanation for this can be given by the fact that firstly only a few data in literature are available and, secondly the baseline concentration of the nets, the net materials and the soap used might have been different. Because of this one can not say that washing is the only determining factor explaining the differences between the literature and the observed data.

**Table 6:** Literature data of laboratory hand washing simulation with “Savon de Marseille” and current study data with “CIPAC washing agent”.

Type of nets	N° ofwash	Current study dataLaboratory hand washing simulation withCIPAC washing agent		LiteratureLaboratory hand washing simulation with“Savon de Marseille”	
		g a.i./kg of net	Overall retention (%)	g a.i./kg of net [authors]	Overall retention (%)
Interceptor®	0	4.72		–	
	1	3.70	78.3	–	–
	3	3.35	70.9	–	–
	5	3.24	68.7	–	–
	15	1.52	32.1	–	–
	20	1.03 or 41.36*	21.9	40* [12]	–
PermaNet®2.0	0	2.00		2.06 [34]	
				1.50 [44]	
	1	1.59	79.7	2.57 [34]	124.8
				1.28 [34]	85.3
	3	1.26	63.1	2.11 [34]	102.4
				0.96 [44]	63.8
	5	1.04	52.1	2.04 [34]	99.0
				0.59 [44]	39.5
	15	0.65	32.5	1.59 [34]	77.2
	20	0.51	25.5	1.20 [34]	58.3
				25* [51]	
Netprotect®	0	1.12		1.95 [34]	
				1.88 [44]	
	1	1.04	92.7	2.00 [34]	102.5
				1.85 [44]	98.3
	3	0.97	86.1	1.97 [34]	100.9
				1.74 [44]	92.2
	5	0.88	78.0	1.92 [34]	98.2
				1.65 [44]	87.6
	15	0.86	76.2	1.71 [34]	87.3
	20	0.72	63.9	1.52 [34]	77.6

A comparison between the literature data and the data obtained in this study is presented. The comparison of these data is based on the overall percent of retention.

* the concentration was expressed in mg a.i./m^2^ of the net.

Coming back to the current study, the retention/release and the concentration of the active ingredient of LNs washed following the laboratory hand washing simulation (WHOPES standard washing procedure with the proposed CIPAC washing agent) were compared with the LNs washed with the domestic washing procedure (ISO 6330∶2000 machine washing).

**Figure 6:**
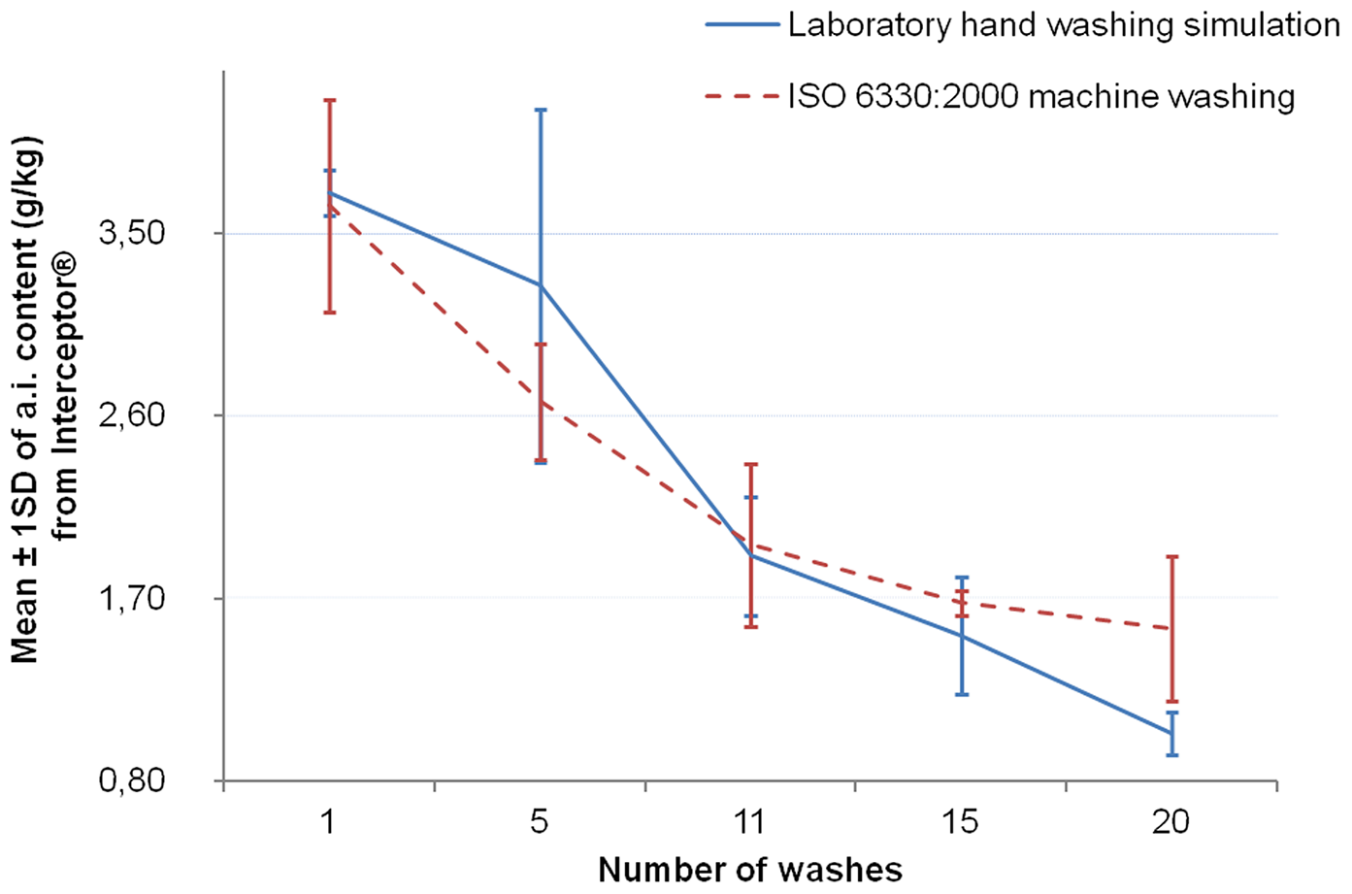
Influence of washing procedures on alpha-cypermethrin content of Interceptor® nets. The mean concentration of alpha-cypermethrin on the net (±1 standard deviation) against the number of washes for each washing procedure is shown.

**Figure 7:**
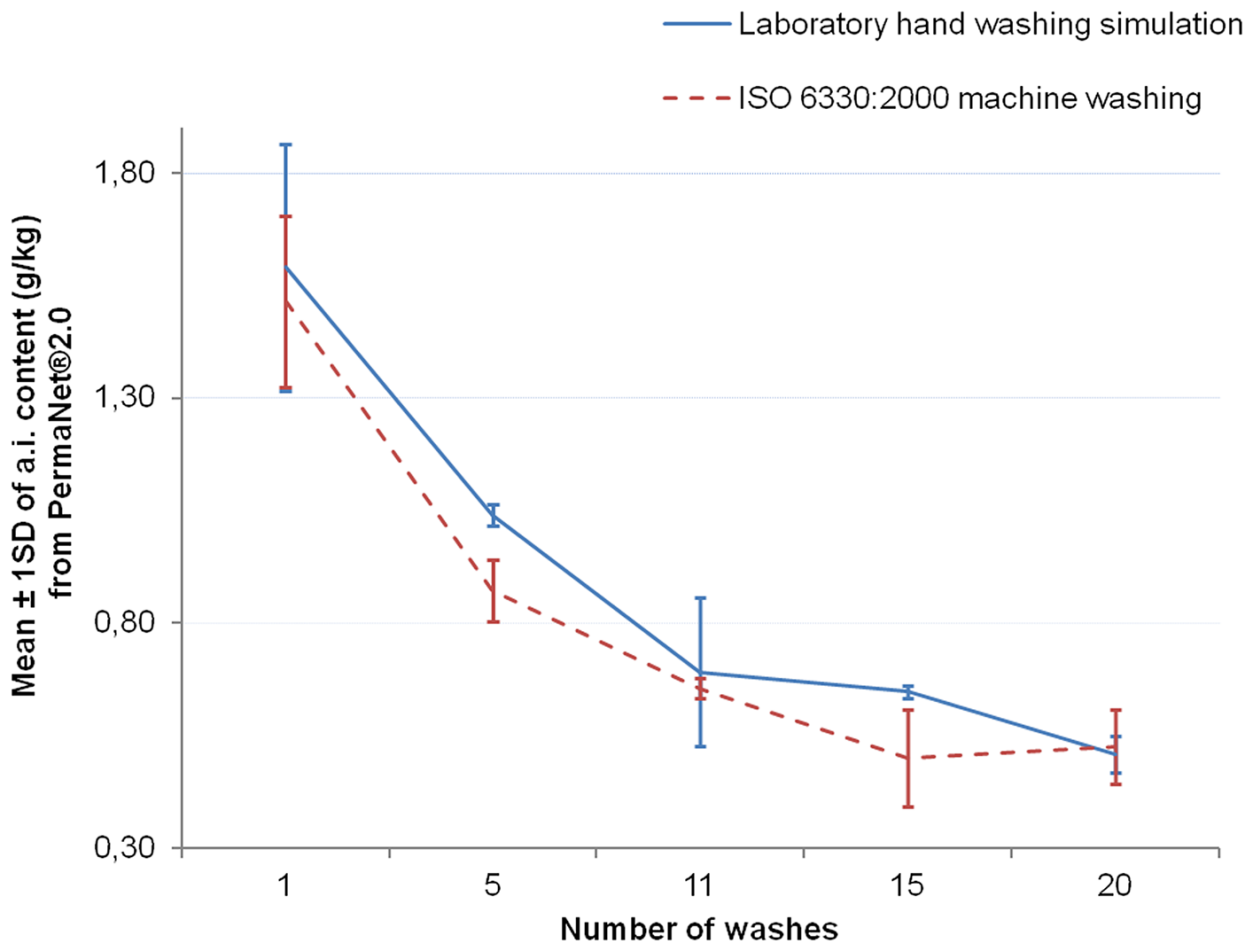
Influence of washing procedures on deltamethrin content of PermaNet®2.0 nets. The mean concentration of deltamethrin on the net (±1 standard deviation) against the number of washes for each washes procedure is shown.

**Figure 8:**
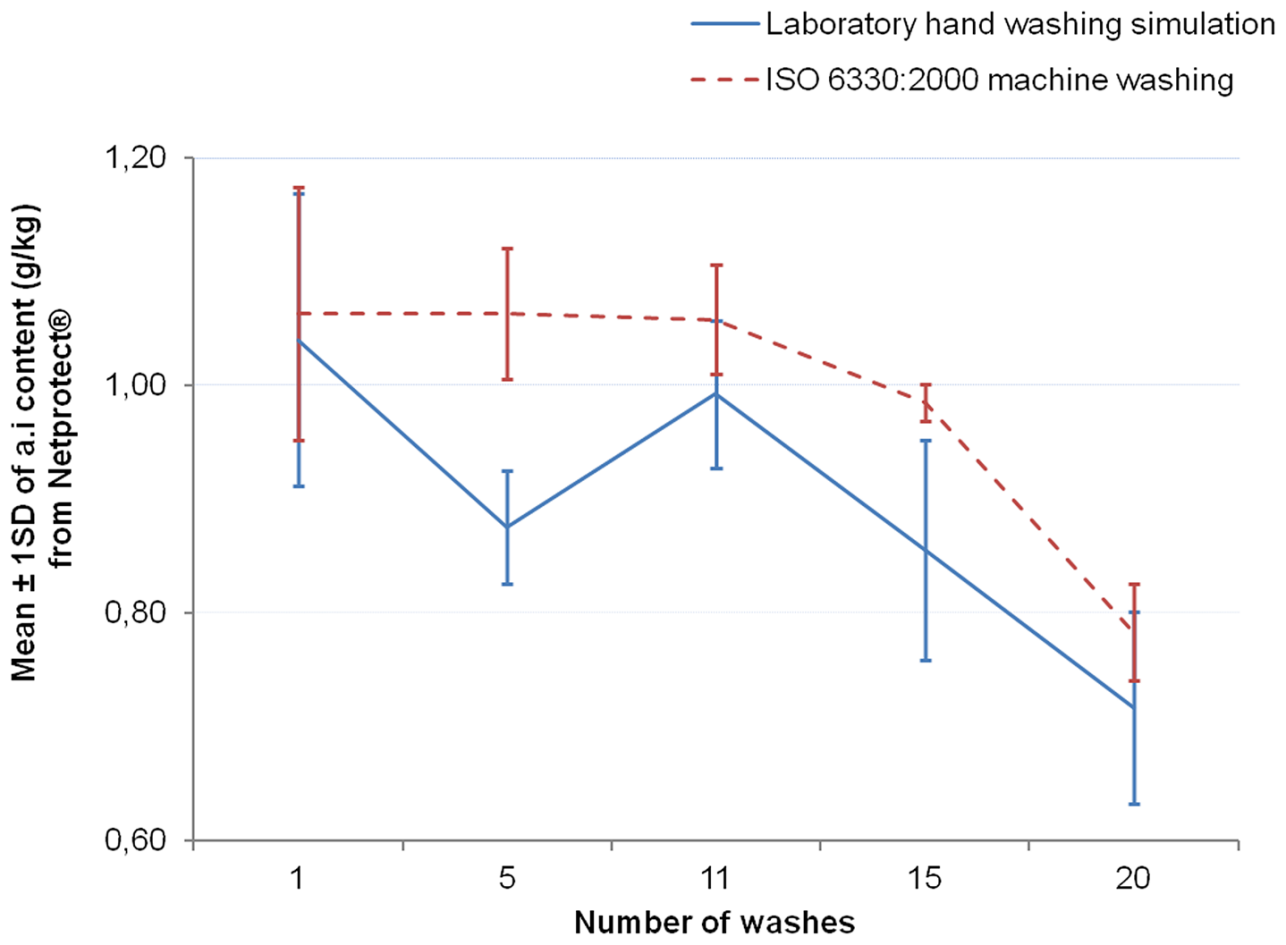
Influence of washing procedures on deltamethrin content of Netprotect® nets. The mean concentration of deltamethrin onto the net (±1 standard deviation) against the number of washing for each washes procedure is shown.

The Figures 6–8 show the graphs of the active ingredient content from the LNs in relation with the number of washes for both washing procedures. Figure 6 and 7 show no difference of the effect of both washing procedures on active ingredient content of Interceptor® and PermaNet®2.0 nets. This was not the case for the Netprotect® nets (Figure 8). For these nets, the concentration of the active ingredient after washing with the laboratory hand simulation procedure was lower than after washing with the ISO 6330∶2000 machine procedure. All these observations were confirmed by statistics shown in Table 7.

**Table 7:** Summary of statistic test for comparison of laboratory hand washing simulation versus ISO 6330∶2000 machine washing (Model I analysis of variance).

Type of Nets	F-test		
	F-value	Df	P-value
**Interceptor®**	1.002	1 and 24	0.237*
**PermaNet®2.0**	4.022	1 and 24	0.056*
**Netprotect®**	11.169	1 and 24	0.003

The same removal of the active ingredient from the coated nets (p>0.05) with both washing procedures and a significant difference of the removal from the incorporated net are shown.

* The dependent variable (active ingredient content) was transformed into logarithm (ln) to fulfill the assumptions underlying the analysis of variance.

So the influence of the washing procedure depended on the type of LNs. As the P-value was more than 0.05 for the Interceptor® and PermaNet®2.0 nets, the laboratory hand washing simulation and the ISO 6330∶2000 machine washing had statistically the same effect on removing active ingredient form those nets. For Netprotect® the P-value obtained from the statistical test (0.001<P<0.01) showed a highly significant difference between the washing procedures. The laboratory hand simulation washing removed more active ingredient from the Netprotect® nets than the ISO 6330∶2000 machine washing. That shows that insecticides coated on the net seem to be removed easily with a soft washing, while this was not the case for the insecticides incorporated into the nets. This confirmed the fact that washing is considered to be a more important loss mechanism for coated net than incorporated nets [40].

**Figure 9:**
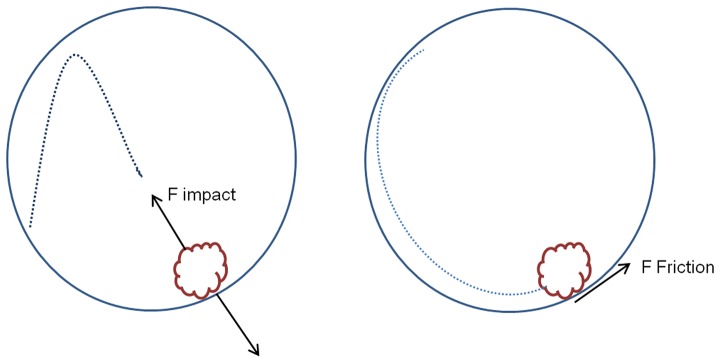
Mechanic forces illustration during the washing process.

The ISO 6330∶2000 machine washing procedure that is considered to be the more stringent [27] (European standard for domestic washing procedure for textiles testing), seems to have less impact compared to the laboratory washing procedure which is considered as a hand simulation washing procedure [41]. So applying the ISO 6330∶2000 machine washing with the IEC A* detergent to the nets underestimates the loss of active ingredient due to washes. At the same time it seems better to wash LNs using this procedure compared to washing by hand. The explanation of this difference might be based on the soap used (chemical action) and/or the shaking process (mechanical action) applied in both methods. The chemical impact might be due to the emulsification actions of surfactants while the mechanical action is caused by the textiles impacting and rubbing against one another [42]. Mechanical action plays an important role in washing process [43], it is seen to be responsible for textile wear; so, it might be also the wear cause of removal insecticide from the net. The washing machine involves important mechanical forces: the normal/impact force and the abrasion/friction force (Figure 9). The abrasion force is related to the drum rotation speed during the main washing. Considering the impact force, it is likely to be higher for the laboratory hand washing simulation than the ISO 6330∶2000 machine washing procedure. This might be why the laboratory hand washing simulation using horizontal shaking affected more the release of the active ingredient from net.

Even if it was found here that the laboratory hand washing simulation removes more insecticide from the nets, it still underestimating the real impact of the washing by hand [19]. Some studies [41], [44] show that the dose of the new CIPAC detergent should be 5.0 g/L in order to reach the expected effect of hand washing. The same studies show that 4.0 g/L CIPAC agent was close to 2.0 g/L Marseille soap in the same washing conditions.

### Effect of Drying: Indoor Drying versus Outdoor Drying with UV Light

**Figure 10:**
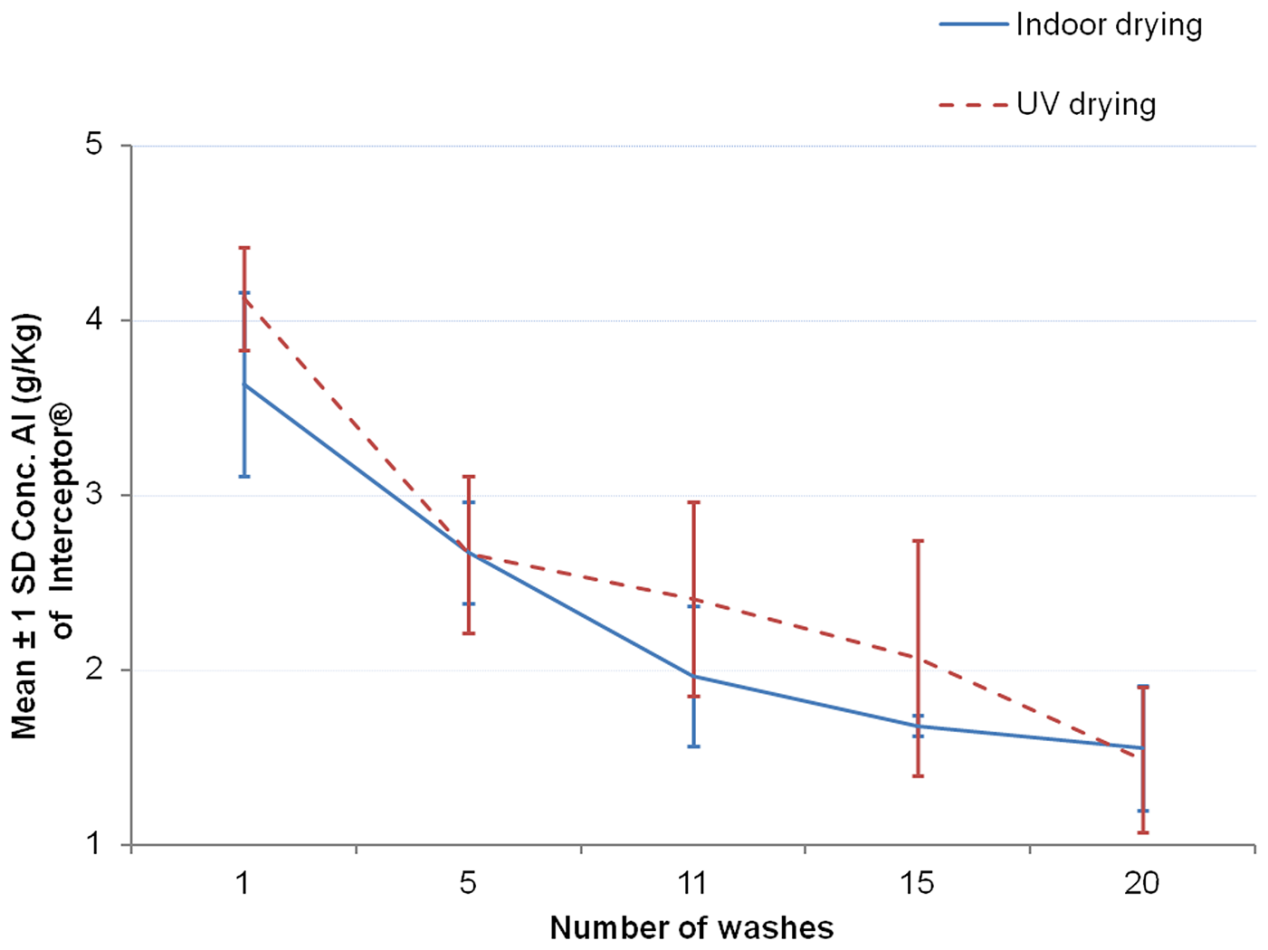
Comparison of drying processes on alpha-cypermethrin content of Interceptor® nets. The mean concentration of alpha-cypermethrin on the net (±1 standard deviation) against the number of washes for each drying procedure is shown.

**Figure 11:**
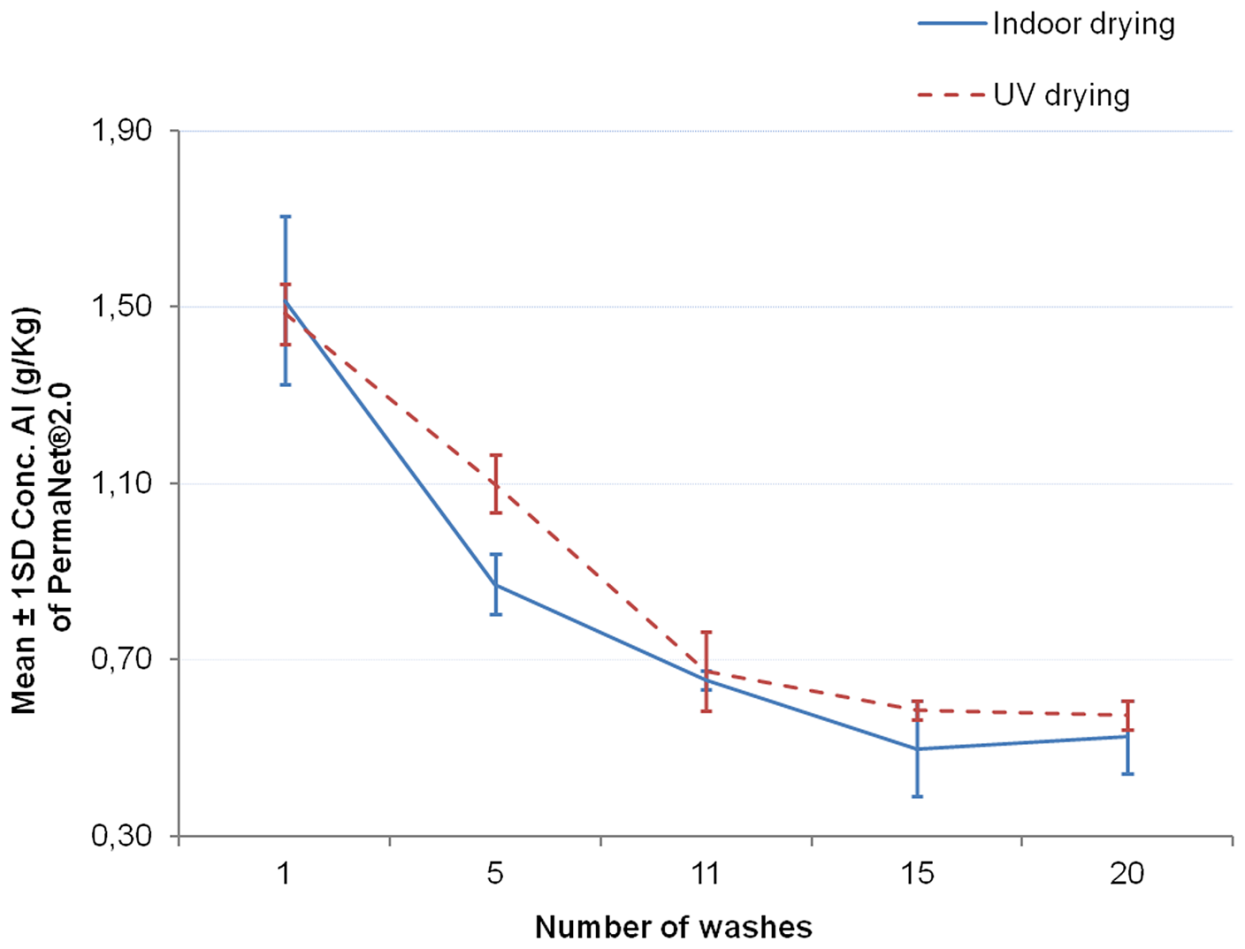
Comparison of drying processes on deltamethrin content of PermaNet®2.0 nets. The mean concentration of deltamethrin on the net (±1 standard deviation) against the number of washes for each drying procedure is shown.

**Figure 12:**
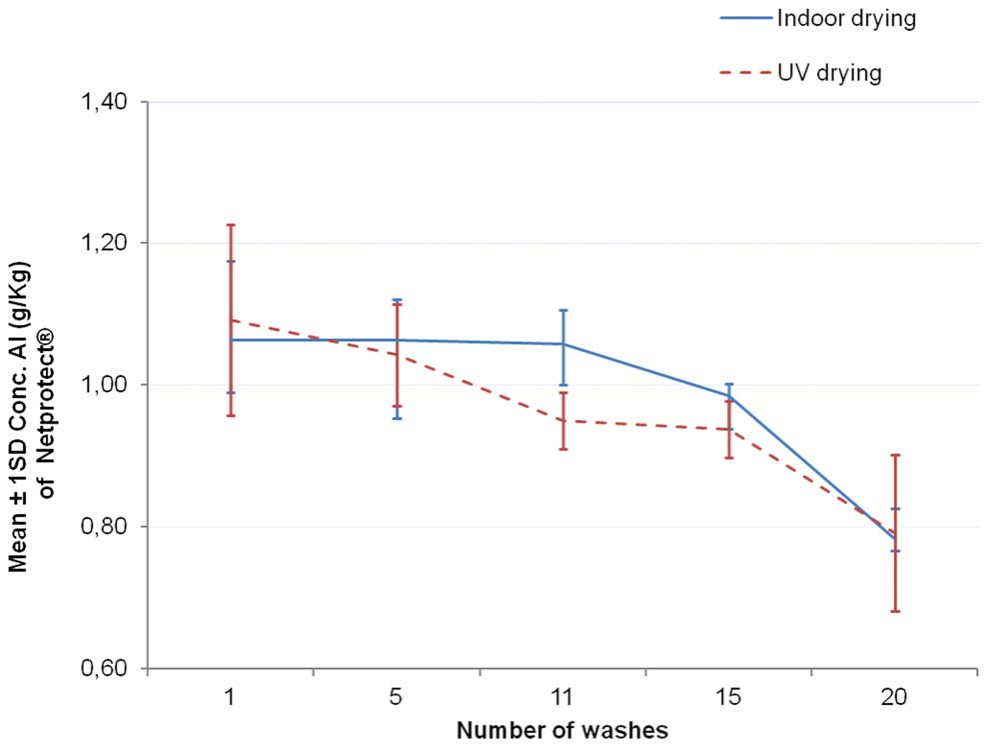
Comparison of drying processes on deltamethrin content of Netprotect® nets. The mean concentration of deltamethrin onto the net (±1 standard deviation) against the number of washes for each drying procedure is shown.

Data obtained from the GC analysis were clustered in graphs (Figure 10, Figure 11, Figure 12) and statistically analyzed with two-way ANOVA using drying processes as fixed factor with 2 levels (indoor and UV drying) and number of washes as fixed factor with 5 levels.

#### Interceptor® nets

The two-way ANOVA model was used to check firstly, whether there is an interaction between the number of washes and the drying method. It was found that even if there was an interaction, it was not statistically significant (F = 0.558; df = 4 and 20; P = 0.696>0.05). That means that a similar effect of the number of washes on the active ingredient content can be expected for both indoor and UV drying.

As for the effect of drying, the data showed no significant difference in the alpha-cypermethrin concentration between the indoor dried samples and the UV dried samples (F = 2.627; df = 1 and 24; P = 0.118>0.05).

#### PermaNet®2.0 nets

The interaction between the number of washes and the drying method was found to be not statistically significant (F = 1.829; df = 4 and 20; P = 0.163>0.05).

Even if an apparent difference is observed from the curve (Figure 11), the two-way ANOVA test revealed no significant difference in deltamethrin concentration between the indoor dried and the UV dried samples (F = 4.230; df = 1 and 24; P = 0.051>0.05).

#### Netprotect® nets

The interaction between the number of washes and the drying method was found to be not statistically significant (F = 0.730; df = 4 and 20; P = 0.582>0.05).

Again two-way ANOVA statistical analysis showed no significant difference in deltamethrin concentration between the indoor dried and UV dried samples (F = 1.078; df = 1 and 24; P = 0.310>0.05).

The fact that we found no significant effect of UV-exposure on the release of the active ingredient from the net [18], [24] might be explained by the fact that the duration and/or the temperature of the exposure during the experiments were not enough to induce the regeneration activity of the insecticide.

Studies about the effect of sunlight on insecticides on LNs give controversial findings. Some state that pyrethroids are degraded when they are exposed to sunlight [23], [45], [46], others [18], [24], [47], [48] contradicted those observations. Taking into account studies for regeneration of insecticides from nets, it can be a hypothesis that the heat from the sunlight might be more responsible for the insecticides dissipation than the UV. A study showed that when LNs were exposed to sunlight (or heat) after washing, the killing effect on mosquitoes increased [15]. This was explained by an acceleration of the insecticide migration to the surface of the net (regeneration). So the next wash removes more insecticide from the surface and in this way the effect of the sunlight on the active ingredient on the net is explained. This is more a consequence of the heating from the sunlight. In this study, only the effect of UV was expected to have an impact on the release of the active ingredient as the temperature in the room (22°C) is less than the average of 40 or 60°C [20], [41], [49] that can involve the regeneration of the active ingredient. In this study it was found that the UV light did not affect the active ingredient content of the nets (independently to the type of net). This can be explained by the fact that all the nets tested are UV protected. Also the intensity of the light may be less than in real outside circumstances.

## Conclusions

The study confirmed that washing affects the concentration of active ingredient independently of the impregnation technology of the net. The total active ingredient content in LNs decreases with the number of washes. Independently of the washing and drying process, coated nets lost 70% of the insecticide content after 20 washes, while incorporated nets lost only 30%. The wash resistance of incorporated nets is higher compared to coated nets. It was also found that in general, the best fitting mathematical model of active ingredient retention/release with washing was the exponential or logarithmic model for coated nets, while the Netprotect® nets did not fit well any of the mathematical models tried out.

The comparison effect of the washing procedures on the active ingredient content remaining in the nets showed that the laboratory hand washing simulation using the CIPAC washing agent at the concentration of 4 g/L released more the insecticide from the mosquito nets compared to the ISO 6330∶2000 machine washing procedure. The washing impact on the LNs depends mainly on the impregnation technologies used. The effect of drying procedures on the release of the active ingredient from each type of net was not statistically significant. This might be due to the efficiency of UV protection technology used by the manufacturers and/or the absence of higher temperatures and/or higher UV intensity.
